# Cooling induces phase separation in membranes derived from isolated CNS myelin

**DOI:** 10.1371/journal.pone.0184881

**Published:** 2017-09-15

**Authors:** Julio M. Pusterla, Emanuel Schneck, Sérgio S. Funari, Bruno Démé, Motomu Tanaka, Rafael G. Oliveira

**Affiliations:** 1 Centro de Investigaciones en Química Biológica de Córdoba (CIQUIBIC)-Departamento de Química Biológica, Facultad de Ciencias Químicas, Universidad Nacional de Córdoba, Ciudad Universitaria, Córdoba, Argentina; 2 Biomaterials Department, Max Planck Institute of Colloids and Interfaces, Potsdam, Germany; 3 HASYLAB at DESY, Hamburg, Germany; 4 Institut Laue-Langevin (ILL), Grenoble, France; 5 Biophysical Chemistry II, Institute of Physical Chemistry and BIOQUANT, University of Heidelberg, Heidelberg, Germany; 6 Institute for Integrated Cell-Material Sciences (WPI iCeMS), Kyoto University, Kyoto, Japan; University of Pittsburgh School of Medicine, UNITED STATES

## Abstract

Purified myelin membranes (PMMs) are the starting material for biochemical analyses such as the isolation of detergent-insoluble glycosphingolipid-rich domains (DIGs), which are believed to be representatives of functional lipid rafts. The normal DIGs isolation protocol involves the extraction of lipids under moderate cooling. Here, we thus address the influence of cooling on the structure of PMMs and its sub-fractions. Thermodynamic and structural aspects of periodic, multilamellar PMMs are examined between 4°C and 45°C and in various biologically relevant aqueous solutions. The phase behavior is investigated by small-angle X-ray scattering (SAXS) and differential scanning calorimetry (DSC). Complementary neutron diffraction (ND) experiments with solid-supported myelin multilayers confirm that the phase behavior is unaffected by planar confinement. SAXS and ND consistently show that multilamellar PMMs in pure water become heterogeneous when cooled by more than 10–15°C below physiological temperature, as during the DIGs isolation procedure. The heterogeneous state of PMMs is stabilized in physiological solution, where phase coexistence persists up to near the physiological temperature. This result supports the general view that membranes under physiological conditions are close to critical points for phase separation. In presence of elevated Ca^2+^ concentrations (> 10 mM), phase coexistence is found even far above physiological temperatures. The relative fractions of the two phases, and thus presumably also their compositions, are found to vary with temperature. Depending on the conditions, an “expanded” phase with larger lamellar period or a “compacted” phase with smaller lamellar period coexists with the native phase. Both expanded and compacted periods are also observed in DIGs under the respective conditions. The observed subtle temperature-dependence of the phase behavior of PMMs suggests that the composition of DIGs is sensitive to the details of the isolation protocol.

## Introduction

Myelin is the membrane system wrapped around neuronal axons that provides neurons with fast signal transmission. Several neurological disorders like Multiple Sclerosis or leukodystrophies are caused by dysfunctional myelin. It is commonly assumed that myelin membranes under physiological conditions contain functional lipid rafts in the form of transiently phase-separated ordered membrane domains [1]. In general, such lipid rafts in membranes emerge at the border of phase coexistence [2–4], and micrographs of monolayers of whole myelin and its purified lipid fraction exhibit stripes and irregular fluctuating shapes, resembling fluctuations near the critical point [5, 6]. For most biochemical-topological studies, for instance on enzyme activity, membrane protein topology, etc., purified myelin membranes (PMMs) are used. PMMs are also the starting material for the isolation of detergent-resistant membrane (DRMs) fractions or detergent-insoluble glycosphingolipid (DIGs) fractions, which are commonly believed to resemble physiological lipid rafts [7–9]. The normal protocols for DIGs isolation involve a step of moderate cooling down to 4°C.

The structure of myelin has been investigated intensively, mainly with X-rays and neutrons [10, 11]. Most of the work has been performed at room temperature or at very low temperatures (below water freezing) to confirm membrane structure preservation in presence of cryo-protectants [12] as well under electron microscopy sample preparation protocols [13]. However, the behavior of myelin under conditions of moderate cooling (4–15°C) has remained vastly unexplored [14, 15], despite its great relevance for DIGs and DRMs isolation, at those temperatures. A few studies on nerve myelin dealt with the effect of heating [16, 17]. In a study on the effect of cooling, wide-angle X-ray scattering (WAXS) measurements revealed that myelin lipid acyl chains remain in a disordered state down to low temperatures, as long as the hydration water is still liquid (above -10°C), and chain crystalline ordering is only observed under conditions of frozen hydration water [18]. No phase coexistence was inferred from those measurements, except for the WAXS work of Chia et al. [19] which suggested a gel-fluid coexistence but could not be reproduced by any other group [18]. To our knowledge there are only two studies about the effect of moderately cooling (up to 4°C) on intact myelin from homeothermic animals [14, 15] and they were both performed with nerve myelin. Regarding PMMs, which are the starting material for DIGs and DRMs isolation, there is much less work [20–22] and studies on the effect of moderate cooling are entirely lacking.

The objective of this work is thus to determine the thermal behavior of PMMs in a wide temperature range covering both physiological and lipid raft isolation temperatures. Small-angle X-ray scattering (SAXS) and neutron diffraction (ND) are used to identify different membrane phases from their characteristic lamellar periods [23–25] assumed in various biologically relevant aqueous solutions that differently stabilize membrane domains [26, 27]. Certain conditions of cooling or of elevated Ca^2+^ levels are found to promote phase separation. The structural results are complemented with differential scanning calorimetry (DSC) measurements, which reveal the thermodynamics of membrane phase transitions. Finally, isolated DIGs are shown to mimic the non-native phase spacings found in the whole myelin in quantitative terms, thus suggesting the similarity of DIGs and the non-native phases of PMMs induced under the respective conditions.

## Materials and methods

### Preparation of PMMs

PMMs were prepared from bovine spinal cord according to Haley et al [28] and it was a gift from *Bustos y Beltrán S*.*A*. abattoir (Córdoba, Argentina) under the supervision of the veterinary of sanity authority. Briefly, the purification protocol consists of several osmotic shocks and direct as well inverse sucrose gradient centrifugations to discard gray matter constituents according to density. After 3 final rinsing steps in water the myelin membranes were lyophilized and stored at -20 or -70°C. Chemicals were of analytical degree, purchased from Merck (Germany), and used without further purification.

PMMs retain myelin biochemical composition [29] as well as TEM patterns (S1 and S2 Figs) of isolated myelin [30, 31]. PMMs also show an electron density profile (S3 Fig) close to that of myelin and purified myelin [21].

### Isolation of detergent-insoluble glycosphingolipid-rich microdomains (DIGs)

The detergent extraction protocol has been described previously [7]. Briefly, 50 mg of PMMs were hydrated, thawed and extracted with 250 ml of 1% TX-100 in TNE buffer (25 mM Tris-HCl/0.15 M NaCl/5 mM EDTA) at 4°C for 30 min with occasional mixing. The TX-100 extracts were centrifuged (13,000 g, 4°C, 10 min) to separate them into detergent-insoluble pellet and detergent-soluble supernatant fractions.

DIGs are enriched in cholesterol, galactocerebroside, cerebroside sulphate, and with low amount of phospholipids. Phosphatidylethanolamine is almost equipartitioned with the soluble supernatant. The major internodal proteins (Folch´s Proteolipid and Myelin Basic Protein) are not found in the DIGs; the paranodal CNPase is also mainly partitioned out of DIGs [7, 8]. S4 Fig in supporting material shows a TEM of DIGs with a pattern different from the one of PMMs.

### Small-angle X-ray scattering (SAXS)

SAXS was measured to determine the lamellar periodicity of PMMs and DIGs multilayers under various conditions.

For PMMs sample preparation, 2–3 mg of lyophilized PMMs were introduced in quartz capillaries (Hilgensberg, Malsfeld, Germany) of 1 mm diameter, and 10 μl of aqueous solutions were added. The solutions were: A) pure (bi-distilled) water, B) physiological Ringer´s solution (145 mM NaCl, 6 mM KCl, 2 mM CaCl_2_, pH 7,4 with 1.5 mM NaHCO_3_/NaH_2_PO_4_ buffer), and C) 25 mM CaCl_2_ in Ringer´s solution. The capillaries were flame sealed and subsequently centrifuged to facilitate the sample hydration. They were then subject to four thawing and cooling cycles (4 to 40°C) and then stored at 4°C.

For DIGs sample preparation, lyophilized DIGs were suspended at 25 mg/ml, warmed up to 45°C to ensure equilibration and filled into the sample holder between two mica plates. A 150 mM NaCl solution was used in replacement to the Ringer's solution and 25 mM CaCl_2_ was employed to induce compaction. The difference in the solutions used for PMMs and DIGs has negligible effect.

SAXS experiments were carried out at the beamline A2 at Hasylab (DESY, Hamburg, Germany) and at the DO2A:SAXS2 beamline at LNLS (Campinas, Brazil), at fixed wavelength of *λ* = 1.5 Å. Scattering signals were recorded either using a linear position-sensitive detector (built by André Gabriel from ILL, EMBL) [32], or a 2D detector (MARCCD 165). In the latter case, radial integration of the Debye-Scherrer rings was performed with the free software Fit2D V12.077 by Andy Hammersley of European Synchrotron Radiation Facility [33]. SAXS intensities are presented as a function of the magnitude of the scattering vector,

$$q=\frac{4\pi }{\lambda }\text{sin}\left(\frac{\theta }{2}\right)$$ (1)

where *θ* is the scattering angle with respect to the incident beam. The corresponding lamellar periodicities *d* then follow from the Bragg equation as

d=2πh/q (2)

where *h* = 1, 2, …, is the peak order.

The number *n* of periodically correlated bilayers under various conditions was obtained by applying the Scherrer equation to the Bragg peak full width at half maximum (*w*) of a Lorentzian curve:

$$n=\frac{2\pi \times 0.88}{d\sqrt{w^{2}-r^{2}}}$$ (3)

where *r* is the instrumental resolution (r ≈ 0.04 nm^-1^).

The diffracting power of native phase was defined [34] by the origin peak of Patterson function:

$$P_{nat}=\sum_{h}\left[\frac{hI_{nat}\left(h\right)}{d^{2}}\right]$$ (4)

where *hI*_*nat*_(*h*)/*d* is the Lorentz-corrected intensity of the reflection of order *h*. The integrated *I*_*nat*_(*h*) was estimated from the areas under the associated Bragg peaks. The fraction of myelin in native phase is expressed as the ratio of the diffracting power *P*_*native*_ to the initial amount (*P*_*native*_ *+ P*_*non-native*_).

The values of SAXS periodicities (*d*), number of correlated bilayers (*n*) and Relative Diffracting Power correspond to the mean of two independent determinations.

### Neutron diffraction (ND) from solid supported membrane multilayers

The ND measurements were performed at the Institute Laue-Langevin (Grenoble) at the beamline D16, with wavelength *λ* = 4.54 Å. Lyophilized PMMs (1 mg) were suspended in 1 ml of bi-distilled water. A 0.5 mL portion of solution/suspension was deposited onto a rectangular (55 x 25 mm^2^) Si(100) substrate with native oxide (Si-Mat, Landsberg/Lech, Germany), which was cleaned by a modified RCA method—ultrasonication in acetone, ethanol, and methanol and subsequent immersing in a solution of 1:1:5 (v/v/v) H_2_O_2_(30%)/NH_4_OH(30%)/water at 60°C for 30 min—[35]. During the process of water evaporation, the amphiphilic molecules self-assemble into planar membrane stacks, aligned parallel with the substrate surface. The PMM-coated wafers were then confined in a sandwich-like liquid cell described elsewhere [36], which brings the multilayers in contact with a thin layer of bulk aqueous medium. The liquid cell, in turn, was inserted into a climate chamber for temperature control. Two different buffers were used: A) 5 mM Hepes + 100 mM NaCl at pH 7.4 and B) the same buffer additionally loaded with 20 mM CaCl_2_. Buffers were based on D_2_O, to enhance the neutron scattering contrast with the hydrogenous PMMs material. Since the membrane multilayers are aligned parallel to the solid support and thus oriented with respect to the incident neutron beam, the measurements involve angular rocking scans, as described earlier for membranes composed of synthetic phospho- and glycolipids [36, 37]. The scattering wave vector component perpendicular to the membrane plane, *q*_z_, assumes the role of *q* in the SAXS experiments (see previous section) and is given as

$$q_{z}=\frac{4\pi }{\lambda }\text{sin}\left(\theta \right)$$ (5)

where *θ* is the incident angle. The lamellar periodicity is then calculated by replacing *q* with *q*_z_ in Eq 2.

### Differential scanning calorimetry (DSC)

A VP-DSC from Microcal, LLC was used with a scan rate of 20°C/h. Generally, several heating and cooling scans (typically from 4 to 46°C) were performed to exclude any influence of thermal history. The concentration of PMMs was 20 mg/ml in all the aqueous suspensions: A) bi-distilled water, B) NaCl 100 mM in water and C) CaCl_2_ 33 mM in water.

## Results and discussion

### PMMs phase behavior

**Fig 1:**
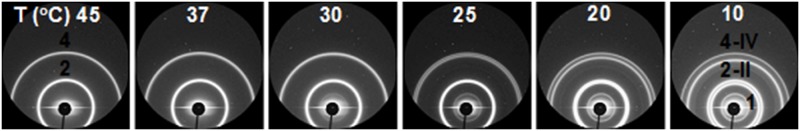
X-ray diffraction pattern of isolated myelin as a function of temperature in Ringer’s solution. At high temperature (37–46°C) two single rings Peaks 2 and 4 are observed. At 30°C the faint smallest peak (1) is observed near the beamstop. At 25°C beam splitting is more easily observed in the peak 4 and IV which is also evident in peaks 2 and II at 10°C.

The phase behavior of PMMs was investigated for various temperatures (4–45°C) and aqueous solutions. SAXS measurements (see Methods section) were carried out to determine the number of coexisting phases under given conditions, as well as their respective lamellar periodicities *d*, the amounts of myelin in the native and non-native phases, perpendicular thermal expansivity *α*_┴_ (K^-1^), and membrane correlation numbers *n*. Fig 1 shows scattering intensities from PMMs in physiological Ringer's solution as recorded with the 2D-detector, featuring Debye-Scherrer rings corresponding to isotropically-oriented multilamellar samples. The intensity patterns obtained at each temperature are reproducible and independent of the thermal history. The appearance of double-rings at low temperatures is characteristic of the coexistence of two membrane phases with different lamellar periodicities.

**Fig 2:**
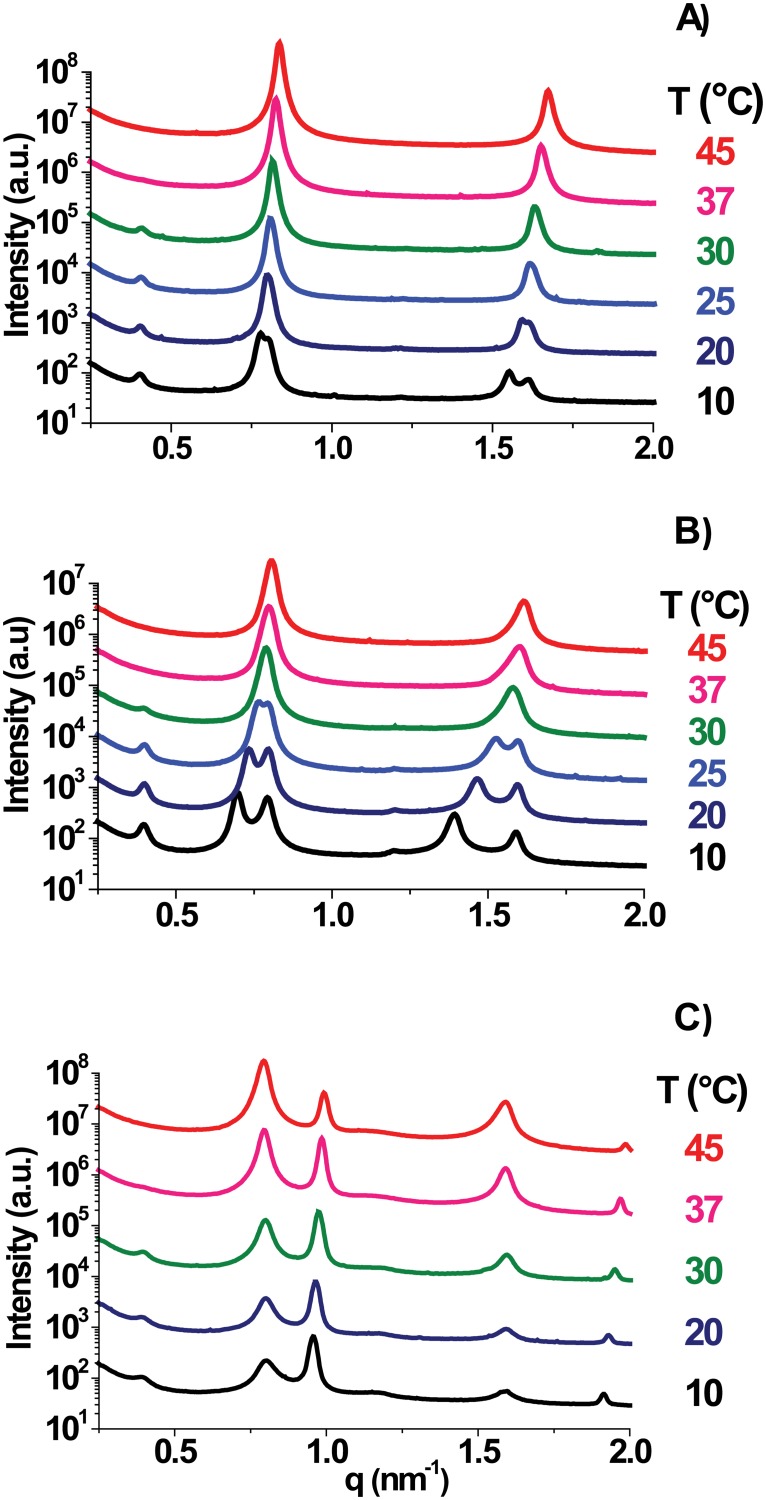
Integrated signals of myelin at different temperatures and in different aqueous conditions. Bi-distilled water (A), Ringer’s solution (B) and CaCl_2_ 25 mM in Ringer’s solution (C). In the case of water dominates a simple pattern and only at low temperature the Bragg peaks splits and shifts to lower *q*. The same happens in the case of Ringer’s solution but at higher temperature. On the other hand, the response to Ca^2+^ goes in the opposite direction in *q* and it is as a jump (all or nothing) instead of a shift.

Fig 2(A) and 2(B) shows radially integrated SAXS intensities plotted vs. *q* for PMMs in bi-distilled water (A) and physiological Ringer’s solution (B) for various temperatures. At high temperature (> 37°C), the scattering intensities exhibit only two major peaks, at *q* ≈ 0.8 nm^-1^ and *q* ≈ 1.6 nm^-1^, respectively, according to the nomenclature by Kirschner [23] corresponding to the peak orders *h* = 2 and *h* = 4 of a double myelin bilayer with lamellar periodicity of *d* ≈ 7.9 nm per bilayer. As the temperature decreases, in both solutions the weak peak of order *h* = 1 becomes increasingly stronger at *q* ≈ 0.4 nm^-1^, which is half the value of the *h* = 2 peak and corresponds to *d* ≈ 15.8 nm, the periodicity of the well-known repeating cell unit of native myelin accommodating two 7.9 nm membranes. Further cooling leads to the splitting of the *h* = 2 and *h* = 4 peaks, associated with the emergence of additional peaks, termed *h* = II and *h* = IV, corresponding to the formation of an additional phase with larger, “expanded” periodicity. The expanded phase appears to have a repeating unit consisting of a single bilayer according to the fact that no *h* = I peak is observed. Its periodicity further expands upon cooling, to 8.2 < *d* < 10 nm as seen from the shift in the *h* = II and *h* = IV peak positions to lower *q* values. Comparison of Fig 2A and 2B reveals that the phase separation occurs already close to the physiological temperature (25–30°C) in Ringer’s solution, while in water a similar splitting of peaks is observed at 20–25°C. As shown in Fig 2C, elevated Ca^2+^ concentration leads to splitting of the *h* = 2 and *h* = 4 peaks for all studied temperatures. The *h* = II and *h* = IV peaks are, however, shifted to higher *q* values, corresponding to the emergence of a “compacted”, lipid-enriched phase with 5.5 < *d* < 6.5 nm, as described in the literature on PMMs and nerve myelin [26, 38]. The native myelin double bilayer of 15.8 nm anyway persists at low T.

**Fig 3:**
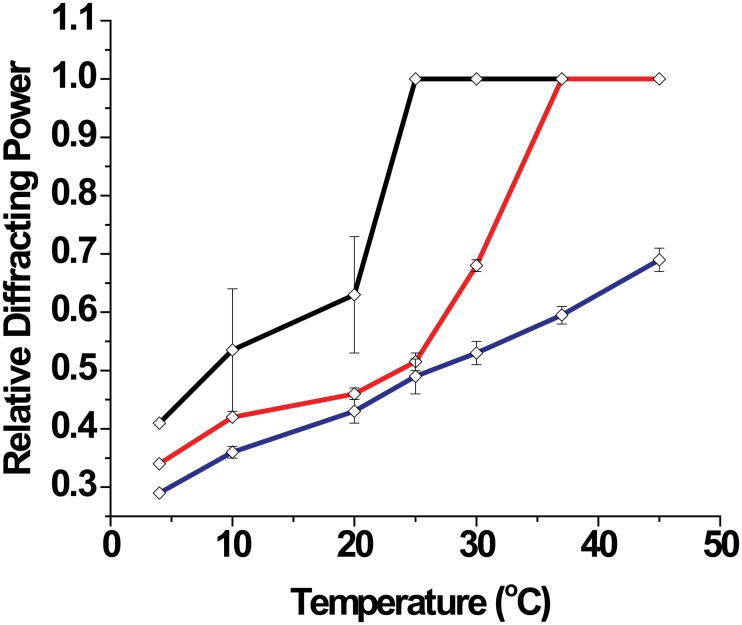
Quantification of the phases as a function of temperature in different conditions. Bi-distilled water (black line), Ringer’s solution (red line) and CaCl_2_ 25 mM in Ringer’s solution (blue line). Data is extracted from the ratio of diffracting power of the native phase to the total amount. Clearly, cooling (as in lipid raft isolation protocol) induces a decay of the native phase fraction and a concomitant increment of the non-native phase (not plotted).

The relative fraction of each phase in dependence of the temperature was estimated from the ratio of the diffracting power of each phase to the initial amount [34]. As shown in Fig 3, when starting from low temperatures heating in all studied aqueous solutions induce growth of the native phase at the expense of the non-native phase. Depending on the composition of the aqueous medium, the non-native phase either disappears above a certain temperature (20–25°C in bi-distilled water, ≈ 25–30°C in physiological Ringer’s solution) or remains partially present in the covered temperature range (at elevated Ca^2+^ concentration).

**Fig 4:**
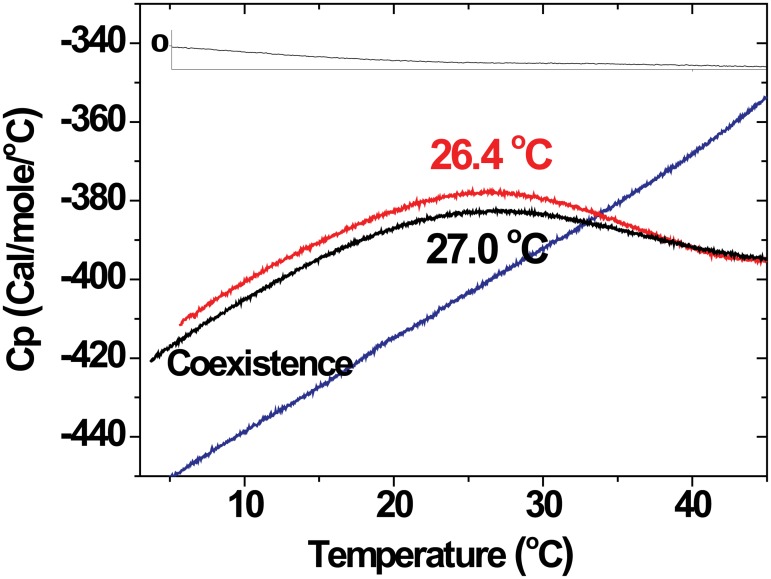
DSC thermograms of myelin (20 Mm) under different aqueous media. In the cases where homogenization takes place–bi-distilled water (black line) and near physiological medium (red line)–a maximum is reached after which a stabilization of the C_p_ is observed near physiological temperature. In the case of high [Ca^2+^] (blue line), a continuous variation of C_p_ according to a phase redistribution of components is observed.

The thermodynamics of the phase transitions was investigated by DSC. Thermal scans of PMMs are shown in Fig 4. For both bi-distilled water and physiological aqueous solution, the heat capacity *C*_p_ exhibits a very broad, endothermic maximum centered at ≈ 26–27°C, near the phase separation temperature. Upon reaching physiological temperature, where only the native phase remains present, *C*_p_ reaches a constant value, indicating the completion of the endothermic transition. This result is consistent with earlier work in similar myelin systems and experimental conditions [39–41]. In contrast, at elevated Ca^2+^ concentration a monotonic increase in C_p_ with temperature is observed in the entire temperature range, possibly suggesting an ongoing transition. This behavior parallels the variation of C_p_ when the myelin is heterogeneous in water and physiological conditions and we speculate that this is due to the redistribution of components as inferred from phase amounts (Fig 3). Although the curves display only small C_p_ variations, they are very reproducible. Earlier calorimetry work on PMMs [39, 41] show a broad endothermic peak at 20–30°C, which has been ascribed to the enthalpies involved in lipid/protein interactions with myelin basic protein (MBP) or Folch´s proteolipid (PLP). In the present work on PMMs we ascribe the broad maxima in *C*_p_ to the membrane phase transitions seen by SAXS. These poorly-defined maxima are probably associated with a phase transition of rather low cooperativity. However, the redistribution of components seems to lead to a change in the heat capacity of the system.

To identify protein-rich and lipid-rich phases, the thermal response of the bilayer thickness was analyzed. For the range of temperatures studied, lamellar periodicities *d* changed linearly with temperature. The perpendicular thermal expansivity (*α*_┴_) of the membrane array was determined from the slopes of the linear functions used to fit the different data, divided by the lamellar periodicities (*α*_┴_ = [∂*d/*∂*T*]*/d*) [42]. In general, protein-rich domains are thicker and less responsive than lipid-rich domains, which are thinner and more responsive [23, 42, 43]. The *q* position of the Bragg peaks associated with the Ca^2+^-induced compacted phase has a response to temperature that is quantitatively accounted for by the well-known negative perpendicular thermal expansivity of lipids undergoing changes in the trans-gauche configuration state, [42, 43] (Table 1). This agrees with the fact that this phase is depleted of intramembrane particles [38]. The same trend is observed in the native phase, but to a lesser extent due to its higher protein fraction [23]. On the other hand, the response of the expanded phase is very strong and cannot be accounted for in terms of perpendicular thermal expansion. Instead, it must be attributed mainly to an increase in the thickness of the interlamellar water layer due to a change in membrane interactions [44].

**Table 1:** Perpendicular thermal expansivity of the different phases in myelin.

Myelin phase	α_┴_ (K^-1^)
Native	-7.2 x 10^−4^
Non-native (expanded)	-5.5 x 10^−3^
Non-native (compacted)	-1.3 x 10^−3^

Periodicity values decay with increments of temperature for all the phases. The expansivities were calculated from the slopes of *d* versus temperature. The linear fit includes all the periodicity values from each phase (native, compacted and expanded), regardless of the ionic conditions.

### Neutron diffraction on planar geometry array

We have previously reported that Langmuir monolayers derived from PMMs at the air/water interface exhibit phase behavior which is qualitatively like that of spontaneously curved PMMs in aqueous suspensions [26]. To elucidate whether the quantitative differences nonetheless observed are related to the absence of one monolayer or rather to the absence of curvature, we investigate planar, i.e., non-curved, PMMs multilayers on macroscopic solid supports.

As a check of the quality of the orientation on the planar support, the mosaicity for the native phase at 37°C (Ca^2+^ free) was 0.17°. After addition of Ca^2+^ the compacted phase had a mosaicity of 0.26°. Both values show a reasonable orientation for hydrated samples [45].

**Fig 5:**
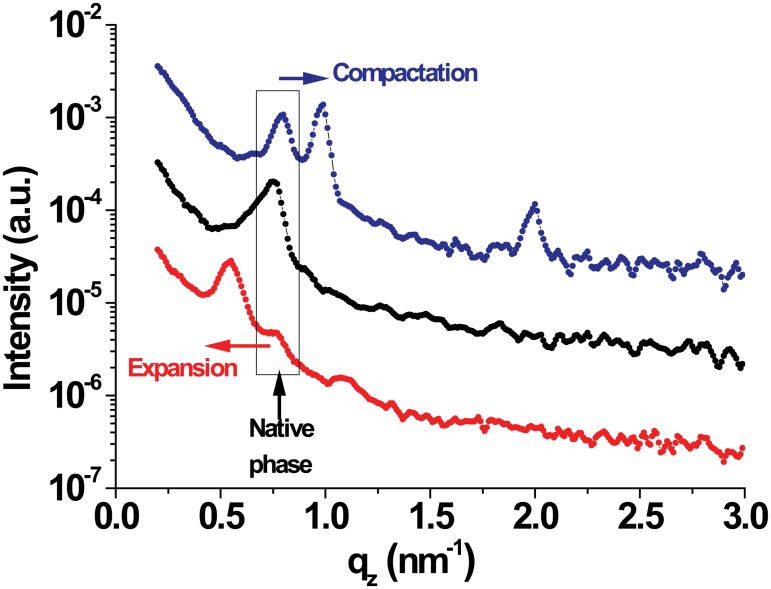
Neutron diffraction signals of multilamellar planar arrays in different media and temperature. The black curve is close to physiological condition. The red curve is the same sample after cooling at 5°C. Two phases are observed with expansion of the spacing. The blue curve is in the presence of high (Ca^2+^) at 37°C; a phase separation takes place in the opposite direction with a compaction.

Fig 5 shows neutron diffraction intensities as a function of *q*_z_ for planar PMMs multilayers in physiological buffer at two temperatures, 37°C and 5°C. As observed in the SAXS experiments on suspended PMMs (see previous section), the single native phase present at 37°C upon cooling splits up into two phases, one of which keeps the native periodicity, *d* ≈ 8.2 nm, while the other one is expanded to *d* ≈ 11.0 nm. The addition of an elevated Ca^2+^ concentration to the physiological buffer again leads to the emergence of a compacted phase, with *d* ≈ 6.4 nm, coexisting with the native phase even at 37°C in agreement with the SAXS results on suspended PMMs (see Table 2).

**Table 2:** Neutron diffraction (planar system) periodicities compared to SAXS on equivalent conditions.

	*d* (nm)—Native phase		*d* (nm)—Compacted phase		*d* (nm)—Expanded phase	
	SAXS	ND	SAXS	ND	SAXS	ND
**37°C–Ca**^**2+**^**-loaded**	7,64±0.07	7.9	6.18±0.02	6.4	-	-
**37°C–Ca**^**2+**^**-free**	7.6±0.1	8.2	-	-	-	-
**4/5°C–Ca**^**2+**^**-free**	7.83±0.07	8.1	-	-	8.9±0.4	11.0

Therefore, as a general conclusion; SAXS, ND and Langmuir monolayers [26] measurements agree in qualitative terms, regarding the kind of phase that is generated in particular conditions. The planar geometry appears to allow further separation to the expanded phase in quantitative terms, as compared to the more self-sealed and curved PMMs. Langmuir monolayers cannot be compared in this regard.

Neutron Diffraction data follows the same trend as SAXS data, displaying analogous behavior. The planar systems display periodicities about 0.2 nm longer; except for the expanded phase in which this effect is maximum reaching a difference of 1.6 nm and a more complete transformation of the native myelin in expanded phase (the native Bragg peak is almost residual).

### Phase behavior of DIGs

**Fig 6:**
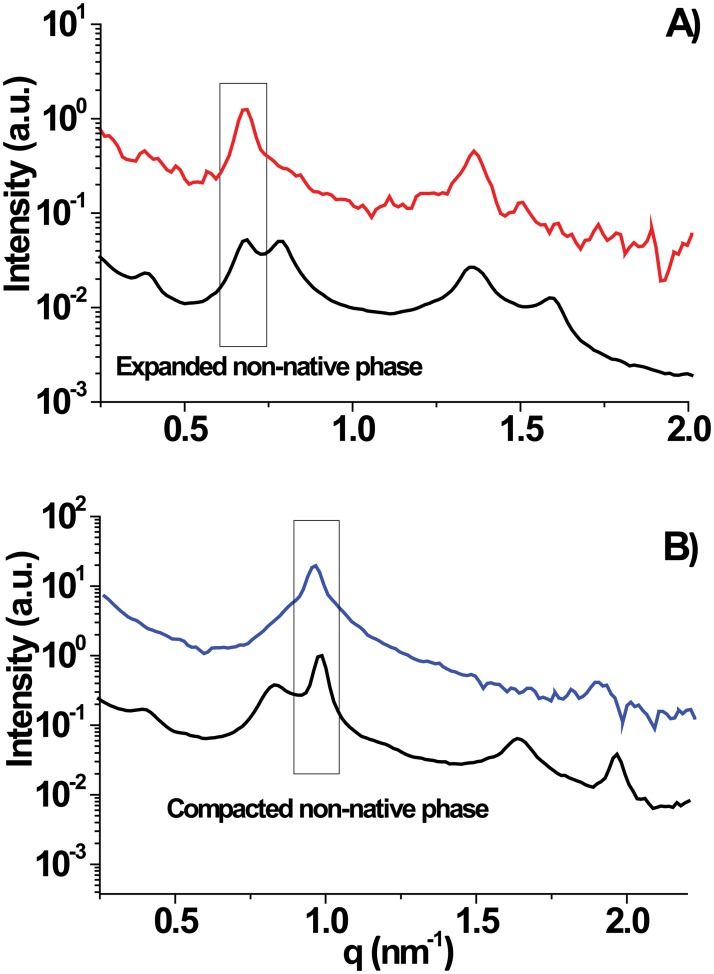
SAXS of DIGs in different ionic conditions at T = 4°C. In the presence of a physiological buffer (A) the first order peaks for DIGs (red dots) and non-native phase from PMMs (black dots) are respectively at 0.66 and 0.67 nm^-1^ indicating lamellar spacings of 9.5 nm and 9.4 (expanded phase). In the presence of CaCl_2_ 25 mM (B) the first order peaks for DIGs (blue dots) and non-native phase from PMMs (black dots) are respectively at 0.96 and 0.98 nm^-1^ indicating lamellar spacings of 6.6 and 6.4 nm (compacted phase).

DIGs were investigated by SAXS at isolation conditions (at 4°C) to compare their periodicity with those of the non-native phases of PMMs. The scattering intensities as a function of *q* are shown in Fig 6. DIGs in physiological ionic strength (5 mM Tris + 150 mM NaCl, Fig 6A), whose periodicity, *d* = 9.5 nm, is close to the *d* = 9.4 nm of the PMMs expanded phase under these conditions. In the presence of elevated Ca^2+^ concentration (Fig 6B) the periodicity of the DIGs fraction is *d* = 6.6 nm, very close to that of the PMMs compacted phase under similar conditions (*d* = 6.4 nm). Thus, we show that the same DIGs fraction can behave as a compacted and as an expanded phase, depending on the ionic milieu conditions.

**Fig 7:**
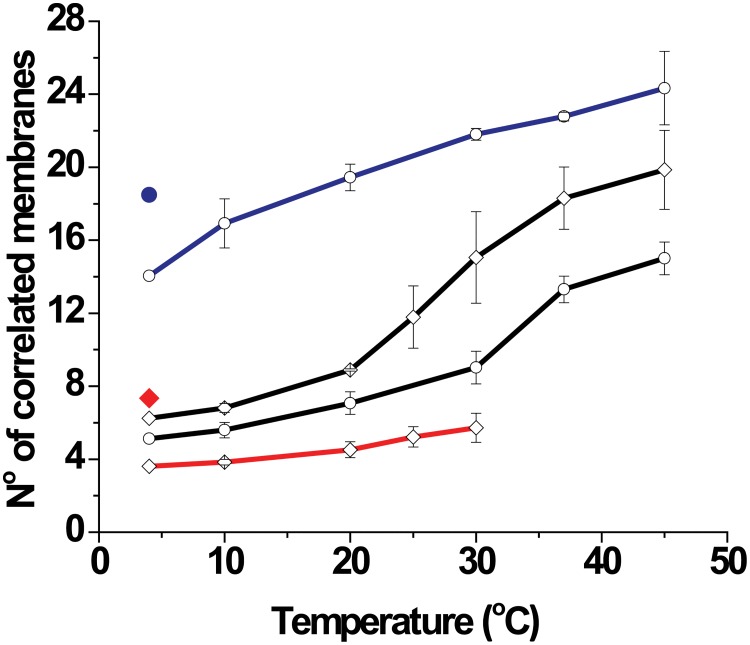
Number (*n*) of correlated membranes for the different phases as a function of temperature for whole myelin (empty) and DIGs (filled). In black is marked the *n* for the native period, in red for the expanded period and in blue for the compact period. In the presence of physiological (diamonds) and high [Ca^2+^] (circles) media.

The similarity of DIGs and the non-native phases of PMMs also manifest in the collective behavior of the membrane multilayers. Namely, as shown in Fig 7, the number *n* of correlated DIGs bilayers, as extracted from the width of the Bragg peaks (see Material and methods), agrees well with the corresponding number for the PMMs non-native phases under the same conditions. For the compacted PMMs phase, *n* ≈ 14–24 showing the highest correlation. The expanded PMMs phase, comprising a thick water layer, is poorly correlated (*n* ≈ 4–6 irrespective of the temperature). Only for the native phase of PMMs *n* exhibits significant temperature-dependence, changing from *n* ≈ 5–6 to *n* ≈ 15–19 upon heating from 10°C to physiological temperature and above. These results agree in general terms with observations of similar myelin systems [14, 21, 38].

There are arguments to think that the presence of detergents modifies the phase diagram of membranes creating or promoting liquid ordered phases [46]. The good point of our approach is that we can detect DIG-like phases in PMMs even in the absence of the detergent. Still there is a possibility that DIGs and non-native phases are not exactly the same [46].

## Conclusion

Classical work pictured nerve myelin as a uniform membrane, phase separation only being obtained after relatively harsh treatments involving osmotic shocks or dehydration [23, 27]. While the expanded and compacted non-native phases of nerve myelin have been identified long ago [38], they were not discussed in the context of putative myelin lipid rafts.

Our group previously demonstrated phase coexistence also in Langmuir monolayers of lipid/protein mixtures derived from PMMs [47, 48]. Nevertheless, the monolayers can also get homogeneous according to lateral packing and ionic media [26] and unexplored variables (temperature, for instance). Therefore, explorations in other systems are mandatory in order to approach new experimental situations. In this sense, PMMs tend to mimic the behavior of nerve myelin in general terms regarding TEM appearance, native spacing, electron density profile and expansion-contraction in response to environmental conditions. [21, 26, 49].

In this work, we clearly show that the cooling of PMMs by about 10–20°C below 37°C induces the phase separation, in a way that is not collectively detectable in nerve myelin. Nevertheless, precursors of phase separation are observed by EM as regions of detachment between the membranes at the extracellular space upon cooling (arrows of Fig 7 in [14]). The presence of phase transitions in nerve membranes at about 25°C was discussed to be related to electromechanical pulse propagation in nerves [50].

The striking similarities in terms of structure and collective behavior of the DIGs and the non-native phases of PMMs at the extraction temperature strongly indicate that the two membranes have very similar constitution. Conversely, this notion implies that the expanded and compacted non-native phases of PMMs are virtually identical [51] and basically differ only with respect to their transverse (interlamellar) interactions under different conditions (see Fig 6). In view of the significant temperature-dependence of fractional weight and, thus, composition of native and non-native PMMs phases, their similarity to DIGs further indicates that the composition of the isolated DIGs significantly depends on the extraction temperature and must therefore be expected to deviate from that of putative rafts transiently forming in myelin at physiological temperature.

Our own studies in Langmuir monolayers strongly support this view [26] of a simple lateral phase separation but with different transverse interlamellar interactions according to the ionic media. Further work in this direction is currently carried out.

The phases shown here are detectable under non-physiological conditions, and one of those conditions (low T) is employed to isolate DIGs, that represent lipid rafts *in vivo* according to some authors. We identify the DIGs as the PMMs non-native phase induced by cooling. Low T is employed during DIGs or raft isolation, not to avoid growth of microorganisms or degradation of the membrane. Rather, cooling alters the membranes and stabilizes DIGs. If DIGs isolation protocol is performed at 37°C, almost no rafts or DIGs are obtained. Low T is used in order to operationally obtain DIGs, but our own results put in question (although not discard) its existence and/or identity at physiological temperature.

## Supporting information

S1 Fig Full field TEM of PMMs at low magnification (60.000x). Pellets of PMMs and DIGs were fixed overnight in 2.5% glutaraldehyde buffered with 0.5 M cacodylate, rinsed three times in distilled water and postfixed in 1% osmium tetroxide buffered with 0.5 M cacodylate at pH = 7. After rinsing, samples were stained in 0.5% uranyl acetate overnight, dehydrated through a graded series of acetone and embedded in epoxy resin. Finally, the thin sections were stained with lead citrate at pH = 12 for 15 minutes. Samples were examined in a JEOL 1220 EXII electron microscope. The micrograph shows a typical pattern of isolated myelin (Kartigashan and Kirschner, 1988; Larocca and Norton 2006). The sample was reconstituted by rehydration of lyophilized PMMs of bovine spinal cord myelin. The characteristic stack of membranes is clearly seen in the image, as well as the rounded shape of some of the stacks.
(TIF)

S2 Fig TEM of PMMs viewed at high magnification (300.000x). The alternation of major dense and intraperiod lines is observed. The major dense lines period is around 13 nm, a little shrink from the normal period as usual, due to artefacts of preparation (Hollingshead and Kirschner 1979).
(TIF)

S3 Fig Electron density profile of PMMs in water at 25°C. The sample was reconstituted from lyophilized myelin powder. The characteristic pattern of myelin consisting in two asymmetric bilayers is observed. Phases are shown in the inset.
(EPS)

S4 Fig TEM of DIGs viewed at high magnification (400.000x). The aspect of the membranes does not match the original one of S1 and S2 Figs.
(TIF)

S1 File Primary data. (RAR)
