# Correction: Cooling induces phase separation in membranes derived from isolated CNS myelin

**DOI:** 10.1371/journal.pone.0189142

**Published:** 2017-11-30

**Authors:** Julio M. Pusterla, Emanuel Schneck, Sérgio S. Funari, Bruno Demé, Motomu Tanaka, Rafael G. Oliveira

The fourth author's name is spelled incorrectly. The correct name is: Bruno Demé. The correct citation is: Pusterla JM, Schneck E, Funari SS, Demé B, Tanaka M, Oliveira RG (2017) Cooling induces phase separation in membranes derived from isolated CNS myelin. PLoS ONE12(9): e0184881. https://doi.org/10.1371/journal.pone.0184881
