# Supplementary figures and images for: Cooling induces phase separation in membranes derived from isolated CNS myelin
Source: PLoS One. 2017 Sep 15;12(9):e0184881. doi: 10.1371/journal.pone.0184881 (PMC5600379; doi:10.1371/journal.pone.0184881)

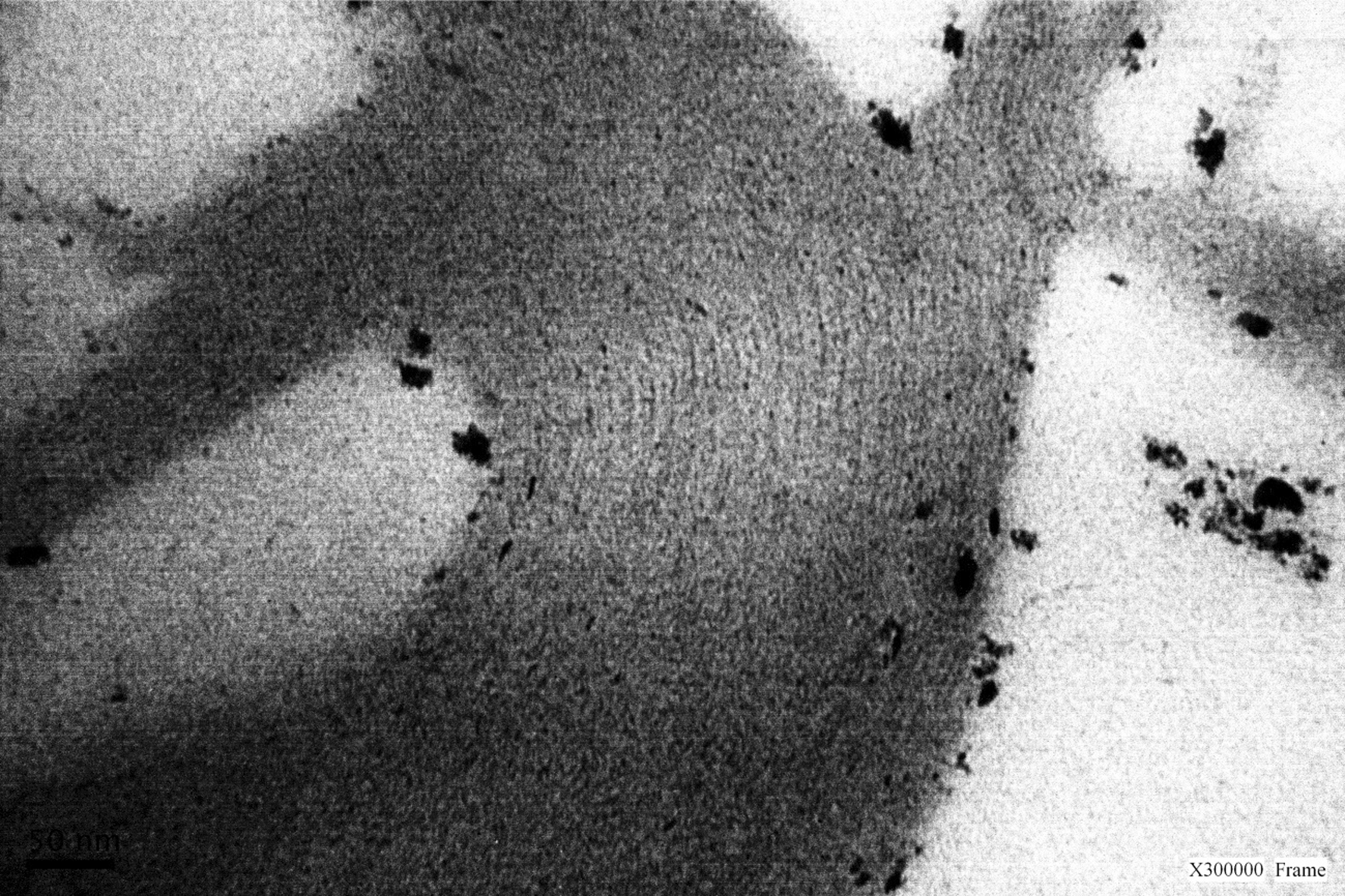

Supplement: S2 Fig — The alternation of major dense and intraperiod lines is observed. The major dense lines period is around 13 nm, a little shrink from the normal period as usual, due to artefacts of preparation (Hollingshead and Kirschner 1979). (TIF) [file pone.0184881.s002.tif]

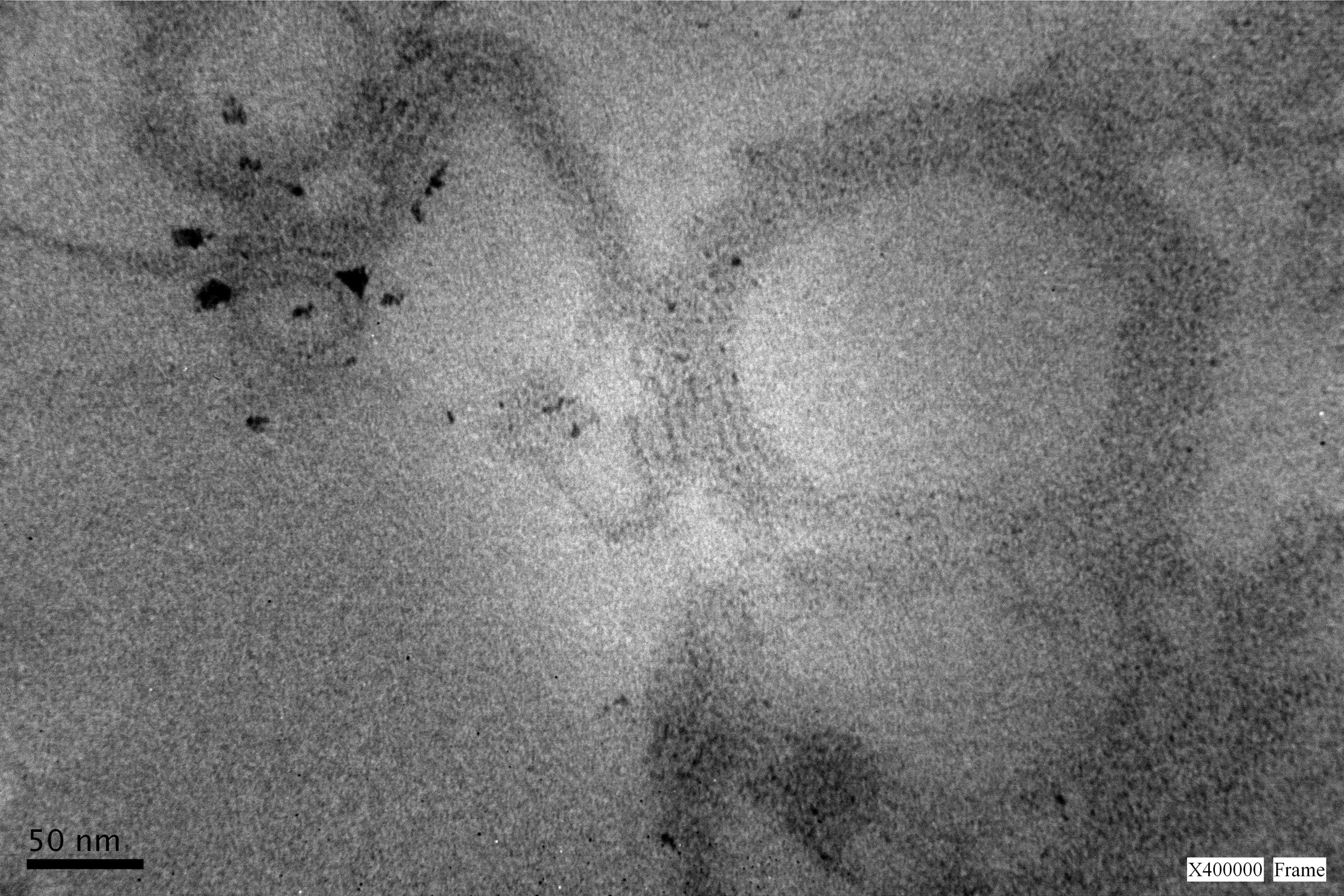

Supplement: S4 Fig — The aspect of the membranes does not match the original one of S1 and S2 Figs. (TIF) [file pone.0184881.s004.tif]
